# Monitoring Sensors for Urban Air Quality: The Case of the Municipality of Lisbon

**DOI:** 10.3390/s23187702

**Published:** 2023-09-06

**Authors:** Rodrigo Sarroeira, João Henriques, Ana M. Sousa, Catarina Ferreira da Silva, Nuno Nunes, Sérgio Moro, Maria do Carmo Botelho

**Affiliations:** 1 ISTAR, Instituto Universitário de Lisboa (ISCTE-IUL), 1649-026 Lisboa, Portugal; rodrigo_sarroeira@iscte-iul.pt (R.S.); sergio.moro@iscte-iul.pt (S.M.); 2 CIES, Instituto Universitário de Lisboa (ISCTE-IUL), 1649-026 Lisboa, Portugal; jpvhs@iscte-iul.pt (J.H.); maria.botelho@iscte-iul.pt (M.d.C.B.); 3 CERENA, Instituto Superior Técnico, Universidade de Lisboa, 1049-001 Lisboa, Portugal; ana.margarida.sousa@tecnico.ulisboa.pt

**Keywords:** air quality sensors, air pollution, health, environmental inequalities, data analysis, municipality of Lisbon, future cities, smart cities

## Abstract

Air pollution is a global issue that impacts environmental inequalities, and air quality sensors can have a decisive role in city policymaking for future cities. Science and society are already aware that during the most challenging times of COVID-19, the levels of air pollution in cities decreased, especially during lockdowns, when road traffic was reduced. Several pollution parameters can be used to analyse cities’ environmental challenges, and it is more pressing than ever to have city climate decisions supported by sensor data. We have applied a data science approach to understand the evolution of the levels of carbon monoxide, nitrogen dioxide, particulate matter 2.5, and particulate matter 10 between August 2021 and July 2022. The analysis of the air quality levels, captured for the first time via 80 monitoring stations distributed throughout the municipality of Lisbon, has allowed us to realize that nitrogen dioxide and particulate matter 10 exceed the levels that are recommended by the World Health Organization, thereby increasing the health risk for those who live and work in Lisbon. Supported by these findings, we propose a central role for air quality sensors for policymaking in future cities, taking as a case study the municipality of Lisbon, Portugal, which is among the European cities that recently proposed be climate-neutral and smart city by 2030.

## 1. Introduction

Improving air quality should continue to be a policy target, as poor air quality is known to be harmful to health. According to the World Health Organization (WHO), air pollution is still one of the largest health threats worldwide. Every year, there are seven million premature deaths caused by air pollution [1]. Regions and cities have been motivated to protect citizens’ health and design and implement plans to improve air quality parameters. One of the Lisbon region’s planned measures for improving air quality [2] is to promote the study of areas with insufficient information and potentially relevant impacts regarding air pollution emissions. To this end, and to fill the spatial gap in air quality measurement, in mid-2021, the Lisbon City Council (CML, Câmara Municipal de Lisboa) installed a new sensor network with 80 air-monitoring stations. Using this new network, and for the first time with this breadth, it is possible to obtain an air data thinner mesh and map the distribution of air quality in the municipality. Therefore, the first objective of this study was to map the spatial distribution of air quality parameters in Lisbon’s urban space. It was thus possible to carry out a comprehensive assessment of air quality parameters with the widest spatial coverage of permanent stations for a time horizon of one year. The second objective was to compare this distribution with the city’s physical space, namely, transport infrastructure and housing density, which are fundamental issues for dealing with environmental inequalities in future cities. The contributions of our work are as follows:

- We characterize the municipality of Lisbon based on the locations of air-quality-monitoring stations, green spaces, road infrastructures, and housing density, with the goal of relating these characteristics to the distribution of air quality parameters.
- We characterize four air pollution parameters: Carbon Monoxide (CO), Nitrogen Dioxide (NO_2_), Particulate Matter 2.5 (PM_2.5_), and Particulate Matter 10 (PM_10_), over a period of one year, starting in August 2021. With the first validated and available data series, the collected data are used to build data models. Then, based on statistics and graphics, we analyse the results and relate them with the main road traffic infrastructures and green areas.
- We adapt the Cross Industry Standard Process for Data Mining (CRISP-DM) methodology [3] to the problem of air inequalities in the municipality of Lisbon’s while instantiating the CRISP-DM phases.

The results of our work can lead to the deployment of new policies geared towards improved air quality sustainability across the municipality of Lisbon and inspire other cities to invest in more advanced sensor policies. The remainder of this paper is organized as follows: Section 2 highlights the relevant literature, Section 3 presents the sensor data analysis methodology, Section 4 characterizes the infrastructures of the municipality of Lisbon relevant to our study, Section 5 introduces the air quality parameters, Section 6 presents the air quality measurement data, and Section 7 analyses the results of our study. Finally, Section 8 provides a discussion and draws conclusions for future cities.

## 2. Theory

Sustainable cities, the digital society, and environmental inequalities are decisive challenges of our time. Cities are decisive spaces in which to tackle climate change and reduce environmental inequalities and health risks. The pertinent theory points out a very complex circular relationship between environmental inequalities and social inequalities and the cumulativeness between both. This relationship can be summarized in three points:

(a) Exposure to pollution: social inequalities are a factor in exposure to different pollution levels, i.e., so-called differential exposure [4,5,6,7,8,9]. The most commonly adopted hypothesis is that the most disadvantaged social categories are more exposed to pollution [4,5]. Although this hypothesis has been confirmed in many places, mainly in semi-urban and rural areas [9], it is not always correct in some large urban regions, wherein highly polluted areas can also have a high cost of living [10,11], which is sometimes caused by planning and the historical development of cities. In this scenario, it becomes essential to study and evaluate the relations between environmental inequalities and health risks in urban contexts and in specific areas within cities.
(b) The effects of exposure to pollution: differential exposure to pollution, along with environmental inequalities, are understood to be factors of social inequalities, especially in the field of health. Studies have pointed out that social inequalities mediate the capacity to combat and prevent the effects generated by equal levels of environmental exposure, i.e., so-called differential susceptibility or vulnerability [4,5,6,7,9,11]. In other words, more favoured social categories have a greater capacity to combat the harmful effects on health resulting from exposure to environmental pollution. Inversely, the most disadvantaged social categories are less able to combat environmental pollution’s harmful health effects.
(c) The relationship between social behaviour and pollution levels: social inequality is seen as a factor that generates different levels of pollution. Recent studies have pointed out that the rich and their consumption and lifestyles are damaging the environment and that the current levels of pollution are mainly caused by wealthy citizens in the most-developed countries, who also have the power to globally spread industrial pollution to other less-developed countries [12,13,14,15].

The territorial distribution of pollution has repercussions on environmental inequalities [12], that is, different social categories are subjected to various pollution levels. In fact, pollution has a harmful effect on human health [16], as environmental inequalities have an impact on a set of health and disease indicators, life, and death [17], which are vital inequalities [18]. To identify the levels of pollution harmful to human health, the WHO defined a set of guidelines serving as a global goal for countries, cities, and governments to work towards in order to improve their citizens’ health; this topic will be considered further ahead [1].

To reduce the main sources of air pollution, energy policies and investments can be enacted and made, respectively, to support an energy transition. Cleaner transport systems, more energy-efficient homes, and better municipal waste management can effectively improve environmental parameters and, consequently, mitigate a population’s exposure to these compounds. Many legal and public policies applied to the transport sector promise to help achieve decarbonization targets. Examples include the shift from gasoline and diesel to lower-carbon fuels or electric vehicles, whether through batteries or fuel cells; an increase in the number of cycling and pedestrian routes; car sharing; and investments in public transport networks. Cities have been making efforts to improve air quality by implementing cycle paths, carrying out road network refinement, and increasing green spaces.

Cities are at a turning point for improving quality of life, inclusion, and equity for those who live, work, and visit them, and this is also because of the harmful effects that pollution causes in terms of health, well-being, resilience, and sustainable development [19,20]. The European Union (EU) has established a need to deeply interlink the European Green Deal and the Digital Transition towards attaining neutral-climate and smart cities. Achieving these objectives is really challenging [21]. To minimize the pertinent impacts and accelerate the energy transition through climate and mobility policies, it is necessary to evaluate the causes of pollution using an effective monitoring system and accurate data, such as those employed in sensor data analysis, to support the decisions made in this regard [22,23,24].

The EU goal of achieving neutral-climate and smart cities by 2030, as in the case of Lisbon, involves facing problems relating to aspects such as air quality, climate change, mobility, transportation, and lifestyles [25,26]. Today, the urban development agenda and its structural investments are based on the achievement of decarbonization, the use of clean energies, and a circular economy. Many public policies at the European, national, regional, and municipal levels arecross-cutting EU priorities of green, digital, just, and inclusive cities [27].

The development of smart cities is supported by the development of sensor networks and the Internet of Things (IoT). This kind of system enables the collection and transfer of information through a defined network with minimal human intervention. This allows for automatic and efficient processing of data.

A smart city uses data collected by electronic sensors to manage resources and monitor interactions within its boundaries. The constant need to collect, store, process, and analyse data requires efficient technological techniques to interact with the big data generated through the process [28].

Several cities are investing in sensor networks. Monitoring air quality (AQ) levels can help to define new policies that aim to reduce air pollution [29] and improve citizens’ quality of life. Europe already has a significant network of air quality sensors, and this number is expected to continue to grow. In Asia, many countries have AQ sensors that are monitored since their air pollution levels have increased. A study was conducted to predict AQ in India using data collected in 23 cities [30,31]. Several cities in China also employ air quality sensors to collect data on pollution parameters [32,33]. Several studies [34,35] have shown that the United States of America also has a dense network of AQ sensors spread throughout its states. In the Lisbon region of Portugal, the authors of [36] analysed the area’s air quality, whereas [37] examined the effects of the economic and financial crisis in relation to the effectiveness of pollution reduction measures, such as the low-emission zone [38,39,40]. Other studies have considered data provided by Copernicus, the European Union’s Earth Observation Programme, to assess the effect of confinement during the COVID-19 pandemic [41], while another study used temporary air quality stations to collect spatial data [42]. In 2022, the Portuguese non-governmental organization ZERO highlighted that pollution levels were higher in three Lisbon locations with heavy traffic (Parque das Nações, Segunda Circular next to Telheiras, and Cais de Sodré) when compared to another location, Lisbon’s Avenida da Liberdade, which also has low air quality [43]. This study analysed data from four temporary stations in Lisbon, located in areas other than the six permanent stations, provided by the national air-quality-monitoring network/QualAr of the Portuguese Environment Agency (APA) [44], showing evidence that the APA’s network is insufficient for characterizing the levels of air quality within the municipality [45]. It is thus relevant to analyse the air parameter data of the new 80 monitoring stations distributed throughout the municipality of Lisbon.

## 3. Sensor Data Analysis Methodology

**Figure 1:**
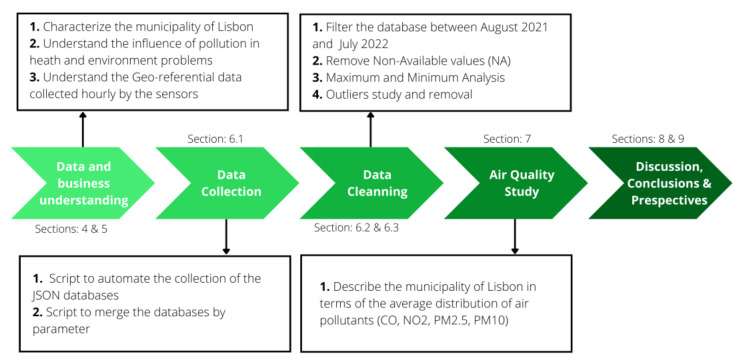
Instantiation of the CRISP-DM methodological approach.

This study adopts the Cross Industry Standard Process for Data Mining (CRISP-DM) methodology, which is widely used in research and practice [3]. It has six phases loosely connected and aims to provide non-restrictive guidance to allow for the success of data mining projects. Figure 1 presents a diagram that integrates the CRISP-DM into our problem. We used the data collected and prepared to build data models based on statistics and graphics to draw recommendations. This study was divided into five phases, which are presented below (Figure 1). The first phase—Business understanding—consisted of the characterization of the municipality of Lisbon in terms of monitoring stations, green spaces, road infrastructure, and housing density. This analysis is presented in Section 4. Then, to understand the data collected by the monitoring stations, the air quality parameters are described in Section 5.

Phase 2—Data Collection—explained the data collection process carried out by the Lisbon City Council’s (CML) monitoring stations. These data are open-source and available at the “Portal Lisboa Aberta” [46]. Section 6.1 presents the process of collecting the databases used to develop the present study.

Phase 3—Data Cleaning—was split into two subphases that are presented in Section 6.2 and Section 6.3. Section 6.2 presents the data description phase that allowed for the identification of errors in the data. A study of the maximum and minimum values and an exploratory analysis of each parameter were carried out. The identified problems in the data were treated in two subphases. The data-cleaning phase is explained in Section 6.3, as well as the analysis and removal of outliers..

In Phase 4, the Air Quality Study, several outputs were generated and analysed, enabling us to draw conclusions about air quality. The charts and maps presented in Section 7 were generated using both Python and QGIS. Maps of the average distribution of air quality parameters are analysed in Section 7.

In Phase 5, Discussion, Conclusions and Perspectives, we used the outputs generated in Phase 4 to draw conclusions regarding the behaviour and spread of the air quality parameters under study (Section 8 and Section 9).

## 4. Case Study: The Municipality of Lisbon

Lisbon is Europe’s westernmost capital city, and its geographical characteristics have positioned it by the Atlantic Ocean and the Tagus River. Lisbon has regular port activity, receiving commercial and leisure maritime transport. Another very relevant characteristic is the airport’s presence within the city. This airport has a high intensity of traffic, and it is currently at the limit of its capacity.

With a resident population of about 544,000, the municipality of Lisbon has lost about 33% of its residents in the last 40 years. In recent decades, housing expansion of the areas surrounding Lisbon has been observed. The city of Lisbon has a population density of 5455 inhabitants per km^2^, which was surpassed by two of its neighbouring cities, Odivelas and Amadora, with the latter having 7210 inhabitants/km^2^, constituting the city with the highest population density in Portugal [47].

**Figure 2:**
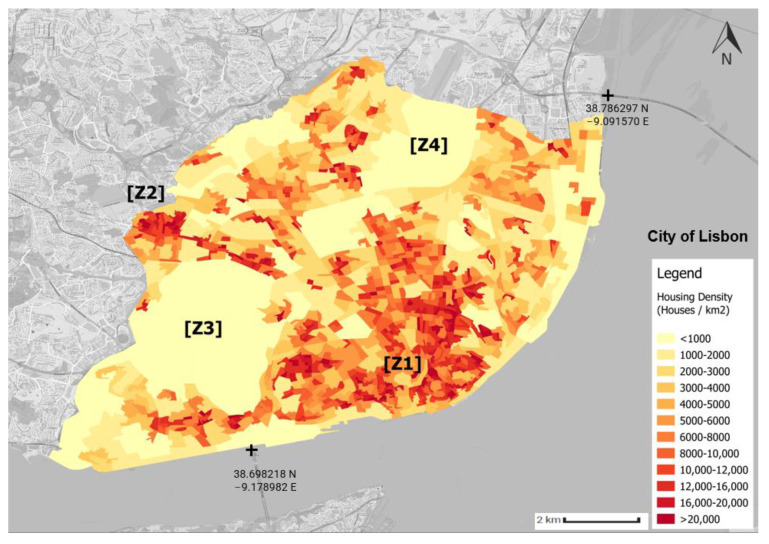
Housing density in the municipality of Lisbon. The source of information is the Censos 2011 [48]. This map was created by the authors of this paper.

Figure 2 presents the housing density in Lisbon. This map was created using statistical subsection census data, which were created in 2011 [48] (2021 census data were not yet available at the time of this study). Each subsection is an area composed of 300 houses. Given this, the area of a subsection is inversely correlated with its housing density, i.e., the smallest subsection is the densest one. The area with the highest housing density is the city centre ([zone Z1)] (Baixa Chiado), and the area with the second-highest housing density is Benfica ([Z2)], with 36,821 inhabitants [49]. Monsanto Natural Park ([Z3)] is an area with 1000 hectares of forest, representing more than 10% of the municipality of Lisbon, and it is the lowest- housing- density zone. The second- largest low- housing- density zone is in the northern part of the municipality, Lisbon’s Airport [Z4].

Housing density is relevant since urban and building infrastructures are important, especially in the cases of NO_2_ and PM_2.5_ [50]. Liu et al. [50] stated that a lower building density promotes the diffusion of PM_2.5_ and NO_2_, thereby mitigating their concentrations.

The housing density on the periphery of Lisbon also has implications for mobility since several citizens work or study outside their place of residence. According to [47], in the Lisbon Metropolitan Area (LMA), the percentage of people who study or work outside the city is between 22% and 33%. Approximately 408,000 people (75% of the resident population) enters Lisbon every day, which means that almost a million people circulate in the city daily.

The movement of the surrounding population towards the city centre is associated not only with the use of public transport but also with a high number of individual modes of transportation. In the LMA, the proportion of the resident population using individual transport for their journeys to work or study is between 43% and 72% [49]. The characterization presented justifies the interest in studying the air quality parameters in Lisbon. Lisbon and its surrounding municipalities have adopted several mitigation measures. The promotion of the use of public transport, the construction of cycle paths [51], the creation of more green spaces, and the encouragement of using electric vehicles [52,53] are examples of some of the actions taken that have been integrated into city’s environmental strategy and policy for the prevention of climate change. Lisbon’s improvement of its population’s environment and quality of life is a work in progress, which is constantly being updated in the city’s agenda. In 2022, the city of Lisbon was considered one of the 100 green cities of the European Union aiming to move towards carbon neutrality by 2030 (EU Mission—Climate-Neutral and Smart Cities) (https://research-and-innovation.ec.europa.eu/funding/funding-opportunities/funding-programmes-and-open-calls/horizon-europe/eu-missions-horizon-europe/climate-neutral-and-smart-cities_en, accessed on 10 January 2023).

**Figure 3:**
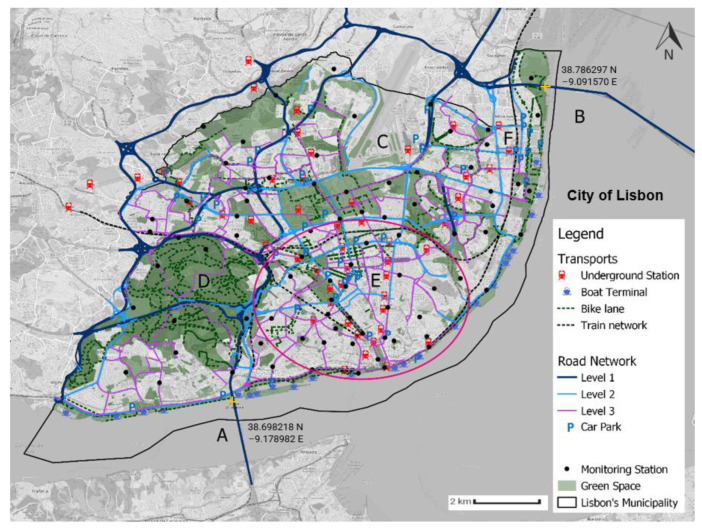
Infrastructures of the municipality of Lisbon. The source of information is the Portal Lisboa Aberta [46]. This map was created by the authors of this paper.

Figure 3 presents the CML’s air-quality-monitoring stations and the basic road infrastructure, green spaces, car parks, and mobility infrastructures of the municipality of Lisbon. This figure was developed using geo-referential data available at the Portal Lisboa Aberta (PLA) [46], and each layer in the map corresponds to a Java Script object notation (JSON) or shape file. These layers were imported from QGIS software 3.32.0 and carefully included to provide relevant information and an understandable depiction of the city. Figure 3 shows the city of Lisbon, which is partially surrounded by the river. To connect with the southern part of the LMA municipalities, Lisbon has two bridges, represented by the letters A and B. The shortest bridge (A) is 2.28 km long and is called the 25th of April Bridge. This bridge usually has high traffic volumes, especially during rush hours. The second bridge, the Vasco da Gama Bridge (B), is 12.35 km long. The area indicated with the letter C in Figure 3 is Lisbon’s airport.

The CML sensor network consists of 80 air-quality-monitoring stations, which are represented by black circles on the map. The distribution of these sensors is generally uniform throughout the city, except for two specific areas: the vicinity of the airport (C) and the central part of the Monsanto Forest Park (D).

The municipality of Lisbon is surrounded by a structured road network that enables individuals to travel long-distance urban routes and supports a second-level main distribution network. This second level enables an inter-sector distribution including the city centre (red ellipse). The main distribution network also has the function of distributing and collecting the traffic from the third-level secondary distribution network, which distributes the traffic within urban sectors connecting to local roads. Travel via bicycle is only allowed in the third- and second-level networks, and it is only allowed in the latter if it is segregated. The car park hotspots of the Saldanha area (E) and Expo (F) are also indicated.

Finally, as Ahn et al. [54] concluded that urban greenways reduce pollution exposure, we decided to analyse the spread of green spaces inside the municipality of Lisbon. The city centre of Lisbon (red ellipse) has few green spaces. The only relevant green spaces in the central part of the municipality are the Baixa Chiado green corridor, the Avenida da Liberdade corridor, and Eduardo VII Park, located near mark E. There are green corridors surrounding the perimeter of the riverbank. The Monsanto Forest Park (D) corresponds to the largest green area in the municipality of Lisbon, with almost 10 km^2^.

**Figure 4:**
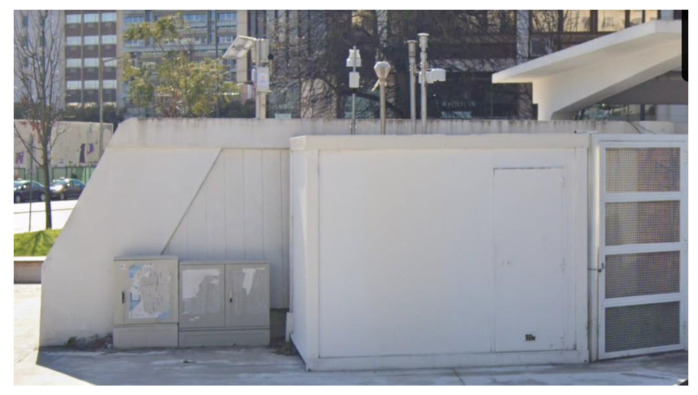
An example of an air-quality-monitoring station in Lisbon (Google maps).

Figure 4 presents an example of an air-quality-monitoring station in Lisbon, where it is possible to observe several sensors used to measure different air quality parameters. The information about the sensors can be found at Portal Lisboa Aberta [46].

## 5. Air Quality Parameters

**Figure 5:**
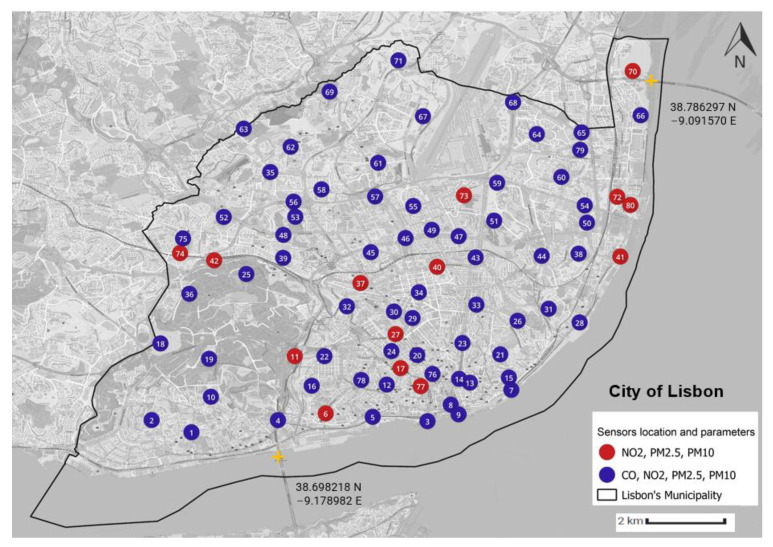
Monitoring stations’ locations and air quality parameters. Each number corresponds to an air-quality monitoring station.

Figure 5 shows the locations of the monitoring stations according to the air quality parameters they measure.

The street address of each monitoring station, identified by its sensor ID, is presented in Appendix A.

### 5.1. Carbon Monoxide

The incomplete combustion of fossil fuels and other organic materials containing carbon is the anthropogenic process responsible for CO reduction [1]. CO can also be obtained from natural sources, such as volcanic eruptions and forest fires [55]. However, in a non-volcanic and urban context, it is expected to be present due to human-related activities.

In cities, one of the main sources of CO is car traffic. Traffic conditions, namely, jams or areas of low traffic speed, where engines are running at high rotations per minute, impact CO concentrations. Furthermore, beyond vehicles with combustion engines, CO also results from electricity production and industrial, commercial, or residential combustion [44]. The WHO provides air quality guidelines for CO, where the CO limit for an 8 h average time is 10,000 μg/m^3^, and the annual average limit is 4000 μg/m^3^ [1]. Prolonged exposure to this gas can cause health problems. The brain, the cardiovascular system, and skeletal muscles are some of the organs and tissues most affected by prolonged exposure to CO.

### 5.2. Nitrogen Dioxide

Nitrogen Dioxide (NO_2_) is a highly toxic gas that results from combustion processes [56] wherein fossil fuels are burnt at elevated temperatures. Industrial, commercial, and residential combustion; combustion engines used for road or marine transport [57]; and manufacturing processes involving the use of nitrogen, such as the production of nitrogen fertilizers, are some methods of producing this gas. NO_2_ can also have a natural origin, i.e., bacterial activity and thunderstorms [58].

In urban areas, transport is the primary source of NO_2_, and emissions from car exhaust primarily come in the form of NO, an unstable molecule that reacts quickly in the presence of oxygen to form NO_2_; in urban areas with high traffic levels, NO_2_ concentrations follow changes in car traffic [1]. The WHO’s maximum recommended level of NO_2_ for an average time of 24 h is 25 µg/m^3^ and 10 µg/m^3^ for an annual average [1].

NO_2_ causes various harmful effects ranging from eye and throat irritation to decreased breathing capacity, chest pain, breathing problems, and damage to the central nervous system and tissues. These effects depend on the concentrations and time of exposure and can cause lung malfunction, which may potentiate a response to allergens in more sensitive individuals [1].

### 5.3. Particulate Matter 2.5 and 10

Particulate matter (PM), such as smoke, dust, dirt, or soot, essentially results from emissions from car traffic, tire degradation, domestic heating, and industrial activities [44]. Natural emissions are also sources of these particles, as is the case with dust from the North African deserts and that resulting from forest fires, which can significantly contribute to increasing PM levels in the Portuguese territory [44].

PM_2.5_ pollutants are among the most hazardous since they constitute tiny particulate matter with a diameter of 2.5 micrometres or less. These particles can pass through the lung barrier and enter the bloodstream, which may result in serious health problems such as cancer and cardiovascular and respiratory diseases [1].

The respiratory system is the most affected by inhalable particles, and their risk to human health depends on their chemical composition and size. Thus, larger PM is usually filtered at the nose and upper respiratory tract level and may be related to irritation and the hypersecretion of the mucous membranes. Smaller particles are usually more harmful, as they are deposited in the functional units of the respiratory system [44]. The WHO recommends an annual limit of PM_2.5_ of 5 µg/m^3^ and a 24 h average limit of 15 µg/m^3^. The annual limit of PM_10′_ is 15 µg/m^3^, for which there is a 24 h average limit of 45 µg/m^3^ [1].

## 6. Air Quality Measurement Data

### 6.1. Data Collection

The air quality measurement datasets are available in JSON format. Each file contains measurements of a specific parameter and location. Our study is based on the analysis of data on four parameters collected from 80 monitoring stations scattered throughout the city in distinct locations. These data were rendered publicly available by the municipality of Lisbon. Each monitoring station has a set of sensors to measure different air quality parameters. Specifically, 320 files were downloaded from the Portal Lisboa Aberta [46]. In this study, only four parameters are considered. The fifth parameter, ozone, is measured in few locations; therefore, we decided to exclude it in this study. Some of the files are empty since not every monitoring station measures every parameter. Each database can be accessed using a Uniform Resource Locator (URL), to which four variables are passed: the parameter, the monitoring station id., and the start and end dates. We developed a script to automate the process of collecting all the database data. The first step consists of a function that generates all the URL combinations of pairs of parameters and a monitoring station’s identification. In the second step, the databases are imported and saved in different folders according to the type of parameter. Each folder ends up with 80 files, with one file per monitoring station. All the files are joined, by parameter, to optimize access to the information.

### 6.2. Data Description

Our study uses data collected by the Lisbon city council (CML) sensor network, the locations of which are presented in Figure 3. The case study focuses on measurements collected between 1 August 2021 and 31 July 2022. The starting date corresponds to the first full month for which the data were rendered publicly accessible after the fieldwork calibration period. The following four air quality parameters, measured in μg/m^3^, allowed for the study of the air quality of the municipality of Lisbon: carbon monoxide (CO); nitrogen dioxide (NO_2_); particulate matter 2.5 (PM_2.5_); and particulate matter 10 (PM_10_). The full dataset comprises 1,984,322 total observations, of which 143,696 are non-available (NA) values, representing 0.57% of the data (Table 1).

**Figure 6:**
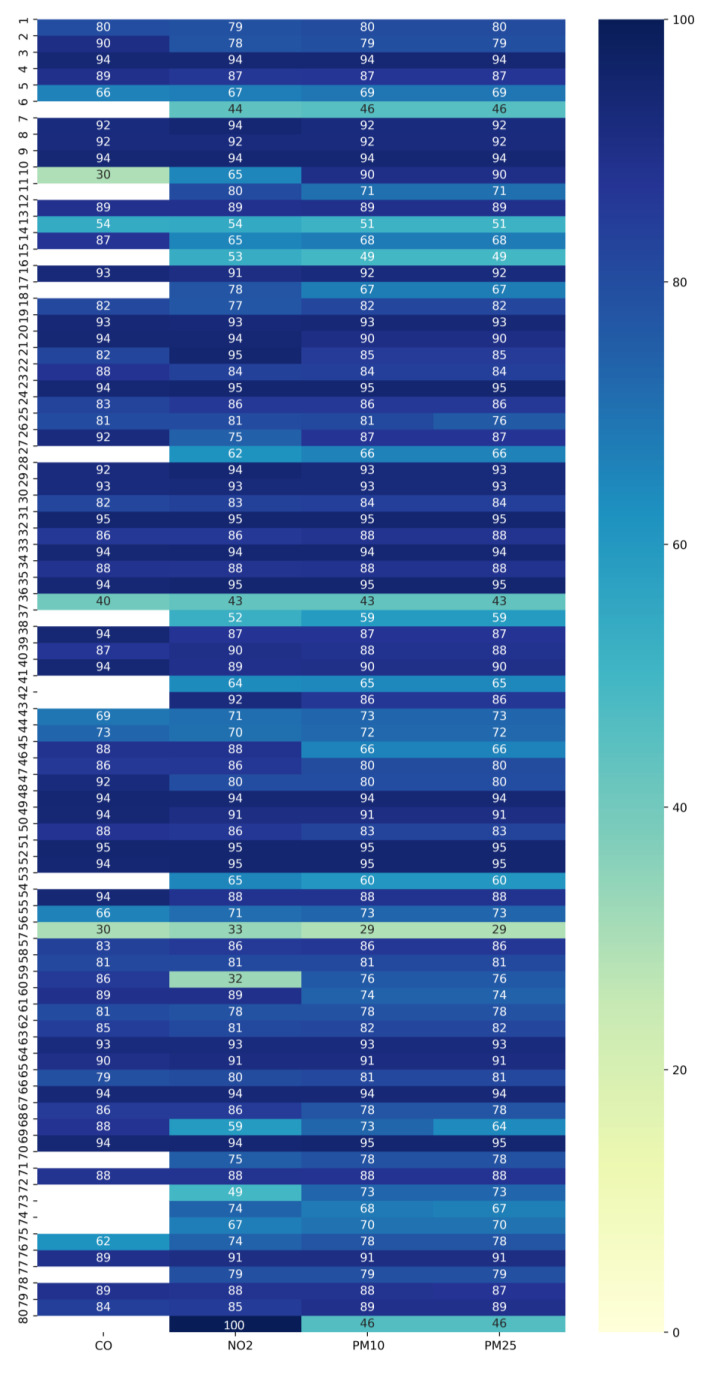
Sensors’ parameter efficiency heatmap.

Figure 6 presents a heatmap containing the sensor efficiencies of the four selected parameters. It is evident that most of the sensors’ efficiencies are above 80%.

### 6.3. Data Cleaning

Table 2 presents the statistics for each parameter. It is fundamental to identify inconsistencies in data. Before conducting this analysis, the missing values, encoded as −99, were removed. Upon analysing the contents of Table 2, several inconsistencies can be identified. Firstly, PM_2.5_ and PM_10_ do not allow for negative values, since these are density measures, but the minimum recorded value is −66. All PM-negative observations were removed from the dataset, given that these are impossible values.

The maximum values, for some parameters, are very far from the average value. This situation is clearly visible for PM_2.5_ and PM_10_. Since these values are so high, they affect the calculated average, thereby biasing the study. To solve this problem, an outlier study was carried out (as presented hereafter).

To find the optimal outlier to remove, boxplots and histograms were generated for each parameter to analyse the distributions of the data. For the four air quality parameters, the following approach was used:

- Calculate Quartiles 1 and 3 (Q1 and Q3);
- Calculate the Inter Quartile Range (IQR = Q3 − Q1);
- Remove all values below Q1 − M × IQR;
- Remove all values above Q3 + M × IQR.

This method was consisted of trying different margin (M) values and analysing the distributions of each parameter through histograms. Several margin values, between one and five, were implemented. When using lower margins, e.g., between one and two, the outlier’s cut-off point affects the distribution curve. To avoid deleting many observations, higher margin values were used. Following this method, only very extreme measurements were deleted from the database, thereby preserving more information. The adopted margin value was four.

**Figure 7:**
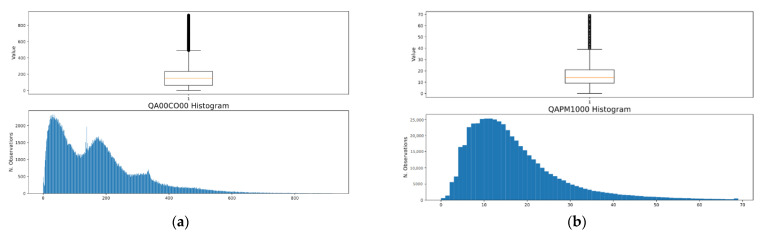
(**a**) Boxplot and histogram of CO measurements after the treatment of the outliers; (**b**) Boxplot and histogram of PM_10_ measurements after the treatment of the outliers.

Figure 7a presents a boxplot and a histogram of the CO measurements. Figure 7b presents a boxplot and a histogram of the PM_10_ measurements. The histograms in Figure 7a,b present the distribution curves of CO and PM_10_, respectively. The right tail of both curves tends to zero, meaning that very few observations were removed during the outlier removal process. As Table 1 shows, the percentages of outliers in CO and PM_10_ are 0.25 and 2.64, respectively. NO_2_ is the parameter that presents the lowest presence of outliers, namely, only 0.03%. PM_2.5_ presents 2.51% outliers in the corresponding data. This method allows for the cleaning of incorrect observations without losing much information.

## 7. Results

**Figure 8:**
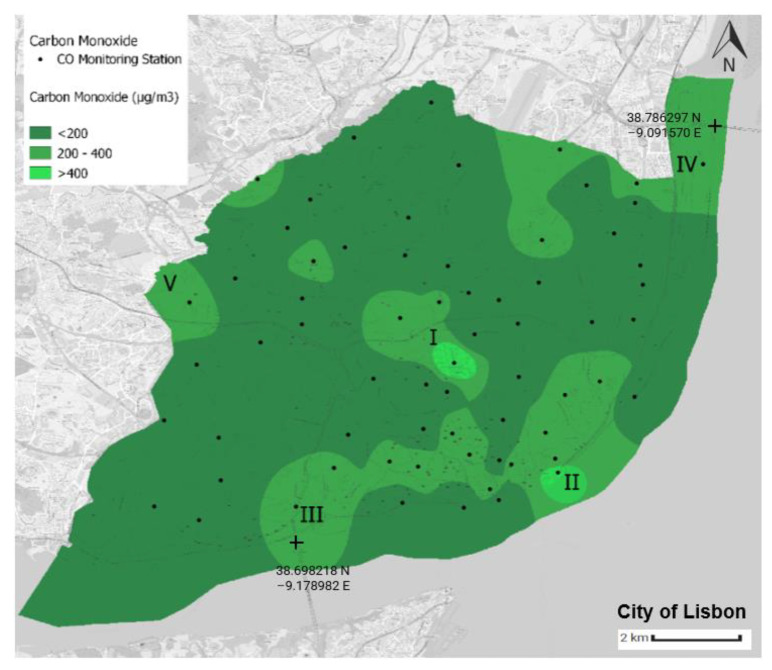
Average annual distribution of CO in the municipality of Lisbon. The source of information is the Portal Lisboa Aberta [46]. This map was created by the authors of this paper.

**Figure 9:**
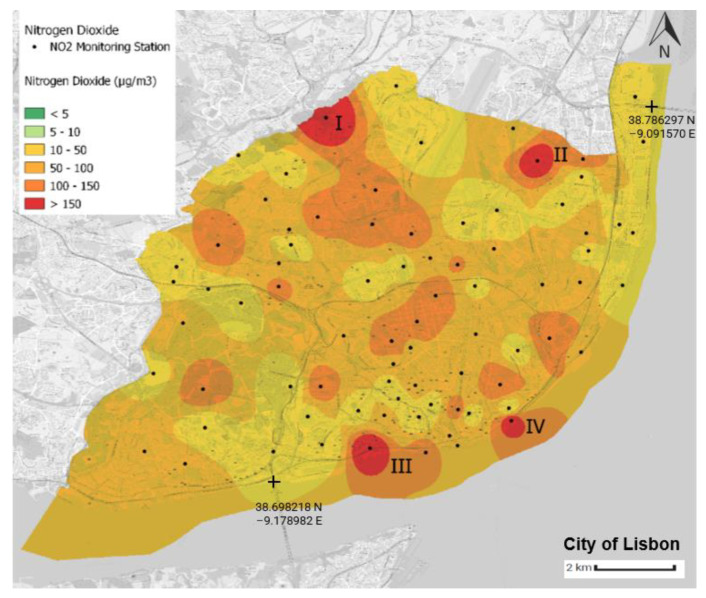
Average annual distribution of NO_2_ in the municipality of Lisbon. The source of information is the Portal Lisboa Aberta [46]. This map was created by the authors of this paper.

**Figure 10:**
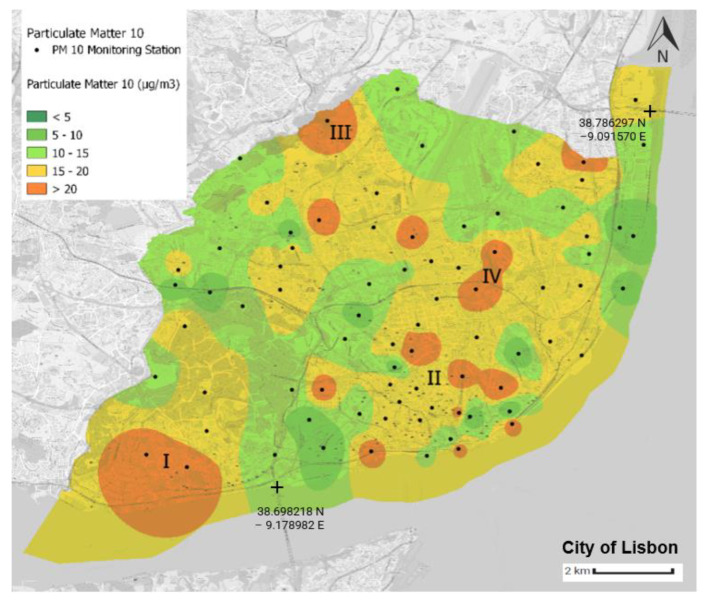
Average annual distribution of PM_10_ in the municipality of Lisbon. The source of information is the Portal Lisboa Aberta [46]. This map was created by the authors of this paper.

**Figure 11:**
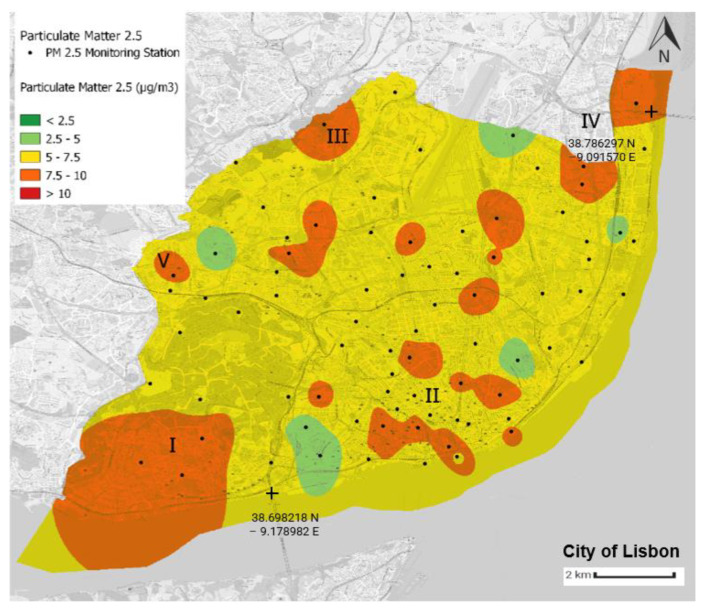
Average annual distribution of PM_2.5_ in the municipality of Lisbon. The source of information is the Portal Lisboa Aberta [46]. This map was created by the authors of this paper.

Using the Python language and the QGIS tool, four air quality yearly average distribution maps in the municipality of Lisbon were generated. The air quality yearly average value for each monitoring station was computed using latitude and longitude values. The scale corresponds to the WHO’s air quality guidelines (Table 3), with green implying that such thresholds were not exceeded (Figure 8, Figure 9, Figure 10 and Figure 11).

### 7.1. Yearly Average Distribution of Carbon Monoxide

Figure 8 presents the mean CO levels estimated for the municipality of Lisbon. During the period under analysis, the average value of all the CO measurements was 175.05 μg/m^3^, and the threshold of 4000 μg/m^3^ was never surpassed in any location.

The monitoring station that presented the highest average value is in Avenida da República (I—Figure 8); this average value was 574.28 μg/m^3^, which is more than three times greater than the global average value. As a study of the traffic in the municipality of Lisbon shows, the surrounding area of this monitoring station is one of the roads with higher levels of traffic [59] (see Figure 5 of the cited study). Using this information, it is possible to associate the CO levels with the traffic density.

The monitoring station of Avenida Infante D. Henrique (II—Figure 8) presented the second-highest average value, 487.11 μg/m^3^, but it did not present high traffic levels, as Figure 5 of the traffic study shows [59]. This seems to indicate that the CO generated in the surrounding area of this monitoring station does not come from vehicle emissions alone. A possible explanation for the source of the CO in the area near this monitoring station is the cruise ship terminal of Lisbon. This terminal is a destination for many large cruise ships throughout the year, and these ships generate extremely high levels of combustion gases. A single cruise ship can generate more pollutant emissions than 12,000 vehicles together [60]. It should be noted that there are cycle paths and wide pedestrian walkways in this riverside area, as it is one of the areas with the greatest tourist and leisure value. Additionally, many residents practice outdoor physical activity in this area.

In addition, the areas with the highest levels of CO pollution are in the central area of the municipality, including the historic and downtown areas. These are areas with a high housing density, and they also have a high share of public transport facilities, including metro and train, both of which are environmentally friendly. Some of the avenues, such as Avenida da República/Saldanha and Avenida da Liberdade, also have bike lanes and wide walkways for pedestrians. The entry zone from the north via the Segunda Circular and the entry zone from the south via the 25th of April Bridge, both of which are level 1 structuring roads, also have higher levels of CO.

### 7.2. Yearly Average Distribution of Nitrogen Dioxide 

Figure 9 presents the mean NO_2_ levels estimated for the municipality of Lisbon. During the period under analysis, the average level of all the NO_2_ measurements was 73.12 μg/m^3^, which is more than seven times higher than the level stipulated in the WHO guidelines. Hence, it is clear that the NO_2_ levels are very high in the municipality of Lisbon. Locations that present a yearly average NO_2_ level above 10 μg/m^3^ exceed the health limits set by the WHO guidelines.

All the monitoring stations surpass the yearly NO_2_ guidelines defined by the WHO. There are thirty-seven monitoring stations that present NO_2_ concentration levels between 10 and 50 μg/m^3^, representing 46.8% of the entire network; 27.8% of the monitoring stations present NO_2_ levels between 50 and 100 μg/m^3^,while 26.6% of the monitoring stations present NO_2_ values between 100 and 150 μg/m^3^. Finally, there are four monitoring stations where the average NO_2_ value was 15 times greater than that stipulated in the WHO guidelines. These four areas are analysed in the next paragraph in more detail.

It is possible to identify important NO_2_ hotspots. For example, close to the Calçada da Carriche (I—Figure 9), a main entrance and exit point of traffic to and from Lisbon and a level 1 structuring road, there are four stations that presented average NO_2_ values between 100 and 150 μg/m^3^. Additionally, the area marked as IV (Figure 9) presented high concentration levels of both NO_2_ and CO (Figure 8, II). This monitoring station, as mentioned above, is close to the cruise terminal of Lisbon. The monitoring station in the Encarnação area (II—Figure 9) presents a yearly average level of 162.11 μg/m^3^. 7.3. Yearly Average Distribution of Particulate Matter 10

Figure 10 map shows the yearly average distribution of PM_10_, where more than half, 55.7% (46 in number), of the monitoring stations surpass the yearly PM_10_ concentration set by the WHO guidelines, i.e., 15 μg/m^3^ (Table 3). There are 16 stations, representing 20% of the network, that present yearly average values above 20 μg/m^3^. These locations are presented in orange and analysed hereafter. During the period under analysis, the average value of PM_10_ was 15.7 μg/m^3^. The areas denoted by roman numerals correspond to the zones where the PM_10_ concentration in the air is higher. Area I, the Belém area, contains two monitoring stations with high average PM_10_ concentrations of 23.06 μg/m^3^ and 20.56 μg/m^3^. Area II (Figure 10), located in the central part of the municipality, contains three monitoring sensors with average values above 20 μg/m^3^. This area contains the two monitoring stations that present the highest average values for PM_10_ in the municipality: 24.16 μg/m^3^ and 23.58 μg/m^3^. The sensor in the Calçada da Carriche (III—Figure 9) presents an average PM_10_ concentration of 23.04 μg/m^3^, the fourth highest in the municipality. The area of Areeiro (IV—Figure 10) has two monitoring stations with levels above 20 μg/m^3^, with yearly average values of 21.12 and 20.44 μg/m^3^. As previously verified for the CO and NO_2_ concentrations, aside from the Belém area, the most problematic areas are in the central part of the municipality, from south to north.

### 7.3. Yearly Average Distribution of Particulate Matter 2.5

In Figure 11, the predominant colour is yellow, which represents areas that present a yearly average PM_2.5_ concentration between 5 and 7.5 μg/m^3^, which is thus above the WHO threshold. There are 51 monitoring stations that present a yearly average between these two levels, representing 64.5% of all the monitoring stations. Only six monitoring stations, representing less than 10% of all the stations, do not surpass the yearly WHO guideline for PM_2.5_. Twenty-two monitoring stations present yearly averages between 7.5 and 10 μg/m^3^. A detailed analysis of the PM_2.5_ yearly average of some of these locations is presented hereafter. The legend’s dark red colour is not present on the map because there are no monitoring stations with yearly averages above 10 μg/m^3^.

By comparing Figure 10 and Figure 11, we can verify that there is a strong correlation between PM_10_ and PM_2.5_ given that the two maps present a similar pattern. This means that these two air quality parameters are not only chronologically but also spatially correlated with each other. This observation makes sense given that these two air quality parameters belong to the same type of particles, with the only difference between them being their diameters. The PM_2.5_ and PM_10_ hotspots overlap in most cases, but the main difference is that the PM_10_ levels do not break the guidelines in as many areas as the PM_2.5_ levels.

## 8. Discussion

Our data analysis shows that the pollution parameters that most exceed the limits defined by the WHO are NO_2_ and suspended particles (PM). Moreover, in the set of four air quality parameters analysed, the areas with the highest levels of pollution tended to be in the zones with the highest housing density, which also coincides with a greater density of public transport and bike lanes (on a smaller scale). This finding allows us to draw two main conclusions. The first is that despite the efforts to reduce pollution in the centre of Lisbon, which include the creation of low-emission zones and an increase in pedestrian zones and bike paths, for specific parameters, the air quality has not yet decreased below the maximum levels proposed by the WHO. The second conclusion is that users of collective means of transport or individual means of transport with low or no environmental impact (such as bicycles and trolleys), that is, active users of environmentally friendly means of transportation, are passively exposed to significant and harmful levels of pollution. This fact generates a discrepancy between environmentally friendly practices and exposure to pollution. A discrepancy between practices and exposure has been previously identified, for example, in England and Wales [61]; however, the cited study mainly focused on comparing the practice of using private combustion vehicles and exposure to the subsequent air pollution, concluding that the rich pollute more and the poor are more exposed. In our study, it was revealed that those who pollute less may be more exposed to levels of pollution that are more harmful to their health since they are directly breathing in the pollution emitted by combustion vehicles when they are walking or cycling. This constitutes an inequality in terms of exposure to pollution since car users are more protected inside their own vehicles. Liao et al. [17] demonstrated that the effects of air pollution increase health inequality (physical discomfort, chronic disease, and self-rated health), harming the most disadvantaged groups to a greater extent since the rich can pay more attention to their health conditions and change their health behaviours. For this reason, it is important to determine who the pedestrians and users of bicycles, trolleys, and public transport are and compare them to car users. In terms of summarizing the results obtained, the following aspects stand out: The distribution of the air quality in Lisbon is heterogeneous and depends on the parameter under analysis. This means that the new network of 80 sensors allows for obtaining a more accurate spatial reading than the national air-quality-monitoring network/QualAr from APA, comprising only six sensors within the municipality of Lisbon. As previous studies have suggested [43], the highest levels of NO_2_ are not registered in Avenida da Liberdade only but also in Calçada de Carriche (a major structural road), Avenida Infante Dom Henrique (next to the cruise port), and Avenida 24 de Julho, among other locations. Therefore, despite obtaining a more reliable characterization of air quality in the municipality, this new network of air quality sensors can still be expanded, either with respect to the number of stations or in terms of the number of sensors per station, since not all parameters are measured at all the stations.

Moreover, this study confirms that there are considerable environmental inequalities in Lisbon. There are some zones in which some of the parameters exceed the annual limits defined by the WHO. The distribution of the air quality parameters also varies depending on the air quality parameter under analysis. Particularly, annual limits for CO were never exceeded at any location, while those for PM_2.5_, PM_10_, and NO_2_ were exceeded. Of the previous three pollutants, NO_2_ is the most problematic. The levels of NO_2_ are higher next to road networks, the cruise port, and in areas with higher population density. For PM_2.5_ and PM_10_, the highest levels were found not only in areas with greater road traffic but also in areas with less population density, such as Monsanto Forest Park. Air quality can be evaluated based on the concentrations of these specific air pollutants using a global Air Quality Index. Future research can be performed by assigning corresponding categories (such as “Good”, “Moderate”, “Unhealthy”, etc.) to indicate the air quality level and potential health risks for citizens. Knowing the places where the air is most polluted can help define a set of policies and measures to help reduce or overcome the pollution problem. For the development of an action plan to minimize pollution, it is necessary to identify the sources of pollution, such as industrial emissions, vehicle traffic, and construction activities. Subsequently, it will be necessary to implement specific, targeted strategies to address each major source of pollution, for example, by introducing stricter emission standards for vehicles, implementing emission reduction technologies in industries, and regulating construction activities. In addition, it will be necessary to promote sustainable forms of transportation, encouraging the use of public transport, cycling, and walking to reduce vehicle emissions. Another method is to create infrastructures that support these modes of transport. In general terms, it is also necessary to strengthen the regulatory framework, establishing and enforcing regulations to control emissions, thus ensuring that enforcement mechanisms are effective. Urban planning and the existence of green spaces will help reduce the heat island effect and improve air quality. Finally, public awareness of these issues, which can be stoked through launching public awareness campaigns about the health risks of poor air quality and the steps that individuals can take to reduce their contribution to pollution, is critical for reducing emissions. This action plan can be executed to reduce the emissions in the specific locations where the levels are higher. To develop this action plan, it is necessary to consider the factors affecting air-quality-monitoring results. In fact, the measurements can indeed vary according to several different factors. While no factor should be completely ignored, some factors might have a larger impact on accuracy and reliability than others. The following is a general ranking of the factors that can influence the results:

(1) Sensor calibration and maintenance: regular calibration and maintenance of monitoring equipment are crucial to ensuring accurate measurements.
(2) Sensor technology: the technology of the sensors used for monitoring plays a vital role, as high-quality sensors are generally more accurate and reliable.
(3) Location of monitoring stations: the location of the monitoring stations determines how representative measurements are in the surrounding area. Stations should be strategically placed in areas with different pollution sources but also free from obstructions that could distort measurements.
(4) Local topography: the local topography, such as valleys and hills, can influence the movement and dispersion of pollutants.
(5) Meteorological conditions: weather conditions, such as wind speed and direction, temperature, and humidity (emulsions formation [62]), can impact the dispersion and concentration of pollutants.
(6) Emission sources: the proximity and intensity of emission sources, such as industrial facilities, vehicular traffic, and construction sites, can significantly affect local air quality.
(7) Sampling time and frequency: the frequency of data collection and the time at which samples are taken influence results.
(8) Data validation and quality control: implementing data validation techniques, such as outlier detection and quality control procedures, is important for identifying and correcting erroneous measurements.
(9) Natural Sources: natural sources, such as pollen and dust, can impact air quality measurements. While not negligible, these might be of less concern in urban areas compared to anthropogenic sources.

These actions can also be coupled with an adaptive traffic-management system [63] to reduce vehicle waiting times and congestion.

## 9. Conclusions and Perspectives

The findings of heterogeneity in the distribution of air quality in Lisbon (covering both more- and less-populated places, including recreational areas, road areas, maritime areas, and historical areas of high cultural and touristic value), which are associated with different sources of pollution, whether anthropogenic or natural, reveal that the implementation of sensor networks is necessary to understand in detail the environmental inequalities of urban territories. Using the data collected by the air monitoring stations, it was possible to map the spatial distribution of environmental inequalities and relate this distribution to the complex socio-morphological characterization of cities. Thus, this study highlights the need to implement air-monitoring stations whose criteria for choosing the location and number of sensors to be installed are comprehensive, given that cities are multifaceted spaces.

Smart cities, particularly those incorporating air-quality sensors and IoT technology [64], are a step forward with respect to facilitating a reliable analysis of the spatial distribution of air quality and, consequently, lead to more assertive policymaking. Smart cities’ air quality is dependent upon the use of sensor data to efficiently monitor air measures. However, while sensors generate enormous quantities of data, their analysis for understanding patterns is more useful if it can contribute to policymakers’ promotion of changes towards the realization of a more sustainable city environment. Additionally, there is a need to maintain such sensors in proper working order, as our analysis was affected by missing values in the available dataset, and to prepare and clean meaningless values.

Our study is limited by 1 year of data, which prevented us from analysing any seasonality effect that may prevail and affect how the municipality of Lisbon should manage its policies according to the season and month. Thus, in the future, we intend to include a timeframe of 3 years to assess seasonality effects. Additionally, measures of meteorological parameters will be included to enable the analysis of their relations with and effects on air quality parameters. Furthermore, supported by the findings of this research, we intend to conduct machine learning and deep learning modelling to predict how air quality metrics can evolve, which is key to taking preventive actions to reduce exposure to bad air quality levels. Accurate air quality predictions might be useful for people suffering from respiratory problems, such as asthma, chronic obstructive pulmonary disease, pulmonary fibrosis, or pneumonia. Knowing which areas will present elevated levels of a determined pollutant beforehand allows citizens to choose which areas to frequent or avoid based on data. The main goal of a smart city is to allow its citizens and policymakers to make data-driven decisions that contribute to a more balanced, inclusive, sustainable, and healthy city.

Another limitation of our study is related to the sensors’ positioning and external influences, which we could not directly remedy. Nevertheless, given the emergence of important studies reflecting advances in sensing and measurements for both sensor placement [65] and the influence of uncertain parameters (e.g., environmental parameters) [66], future studies should be proposed to the CML that would enable the exploitation of these recent advances for the better use of sensing technologies.

The boundary of this study is the municipality of Lisbon since it was the first to install a dense air-quality-monitoring network in Portugal. The methodology can and should be applied to other geographic areas where air-quality-monitoring networks will be installed. In the future, supported by both data science and statistical analysis, a further step will be to evaluate the policies of the municipality of Lisbon regarding the realization of a European neutral-climate and smart city by 2030. Monitoring sensors for air quality, in Lisbon as in other cities, is a fundamental scientific and political strategy to help future cities achieve higher quality of life, inclusion, resilience, and sustainable development. The expansion of the air quality sensor network in cities [67], their maintenance, and investment in support policymaking are decisive paths towards better future cities. However, when deploying sensor networks and capturing sensor data, one should also highlight the imperative of ensuring users’ data privacy protection [68].

## Figures and Tables

**Table 1:** Parameter information, characterization, and data.

Parameter	Unit	Size	N. Sensors	NA%	Outliers %
CO	μg/m^3^	467,057	65	0.17	0.25
NO_2_	μg/m^3^	479,646	80	2.18	0.03
PM_2.5_	μg/m^3^	518,370	80	0.00	2.51
PM_10_	μg/m^3^	519,249	80	0.00	2.64

**Table 2:** Parameters’ statistics before (a) and after cleaning (b).

Parameter	Min (a)	Mean (a)	Max (a)	Min (b)	Mean (b)	Max (b)
CO	0.0009	174.96	10,025.75	0.0009	172.05	925.06
NO_2_	1.00	73.33	8122.00	1.00	73.12	396.00
PM_2.5_	−66.00	10.88	52,852.00	0.00	6.94	29.00
PM_10_	−66.00	24.53	99,096.00	0.00	16.54	69.00

**Table 3:** WHO yearly air quality guidelines.

Parameter	Period	Limit Value
CO	1 year	4000 μg/m^3^
NO_2_	1 year	10 μg/m^3^
PM_2.5_	1 year	5 μg/m^3^
PM_10_	1 year	15 μg/m^3^

## Data Availability

The data used for this study is publicly available at Portal Lisboa Aberta, CML, https://lisboaaberta.cm-lisboa.pt/index.php/pt/, accessed on 2 October 2022.

## Appendix A

**Table A1:** Monitoring stations’ addresses (based on Portal Lisboa Aberta [46]).

Sensor ID	Address	Sensor ID	Address
1	Calçada da Ajuda	41	Jardim do Braço de Prata
2	Restelo—Rua Gonçalo Velho Cabral	42	Travessa de Francisco Rezende
3	Cais do Sodré	43	Avenida Almirante Gago Coutinho
4	Alcântara—Rua dos Lusíadas	44	Avenida do Santo Condestável
5	Avenida 24 de Julho	45	Rua Frei Carlos
6	Avenida Infante Santo	46	Entrecampos
7	Santa Apolónia—Avenida Infante Dom Henrique	47	Avenida dos Estados Unidos da América
8	Baixa—Rua do Ouro	48	Avenida Lusíada
9	Praça do Comércio	49	Avenida de Roma
10	Alto da Ajuda—Rua Sá Nogueira	50	Chelas—Rua Dr. José Espirito Santo
11	Avenida de Ceuta	51	Avenida José Régio
12	Rua de São Bento	52	Avenida Lusíada / Qta da Granja
13	Rua Damasceno Monteiro	53	Rua Lúcio de Azevedo
14	Praça Martim Moniz	54	Avenida Marechal Gomes da Costa
15	Campo de Santa Clara	55	Avenida do Brasil
16	Cemitério dos Prazeres	56	Avenida General Norton de Matos
17	Jardim Botânico	57	Campo Grande—Museu da Cidade
18	Parque de Campismo de Lisboa	58	Jardim Professor António Franco
19	Monsanto—Alameda Keil do Amaral	59	Parque da Vinha—Estação Meteorológica
20	Avenida da Liberdade—Rua Manuel Jesus Coelho	60	Olivais Sul—Quinta Pedagógica
21	Rua dos Sapadores	61	Quinta das Conchas—Avenida Maria Helena Vieira da Silva
22	Campo de Ourique	62	Estrada do Paço do Lumiar
23	Avenida Almirante Reis	63	Estrada Militar
24	Rua Braamcamp	64	Alameda da Encarnação
25	Monsanto—Parque Ecológico	65	Avenida Doutor Alfredo Bensaúde
26	Parada Alto de São João	66	Rua Ilha dos Amores
27	Marquês de Pombal—Alameda Edgar Cardoso	67	Rua Vasco da Gama Fernandes
28	Beato—Avenida Infante Dom Henrique	68	Laboratório de Bromatologia e Águas
29	Avenida Fontes Pereira de Melo	69	Calçada de Carriche
30	Avenida António Augusto de Aguiar	70	Rua Chen He
31	Largo da Madre de Deus	71	Estrada Militar às Galinheiras
32	Rua de Campolide	72	Rua Mário Botas
33	Largo do Leão	73	Rua Alferes Malheiro
34	Avenida da República	74	Rua da Venezuela
35	Praça São Francisco de Assis	75	Alameda Pedro Álvaro Proença
36	Estrada de Monsanto	76	Restauradores—Avenida da Liberdade
37	Praça de Espanha	77	Rua da Atalaia
38	Marvila—Rua Pedro de Azevedo	78	Jardim da Estrela
39	Estrada de Benfica	79	Avenida Doutor Francisco Luís Gomes
40	Avenida João XXI	80	Rua Nau Catrineta
